# Adipose Stem Cells Promote Smooth Muscle Cells to Secrete Elastin in Rat Abdominal Aortic Aneurysm

**DOI:** 10.1371/journal.pone.0108105

**Published:** 2014-09-22

**Authors:** Xiaohong Tian, Jun Fan, Miao Yu, Yu Zhao, Yan Fang, Shuling Bai, Weijian Hou, Hao Tong

**Affiliations:** 1 Department of Tissue Engineering, College of Basic Medical Sciences, China Medical University, Shenyang, Liaoning, P. R. China; 2 Department of Plastic Surgery, Shengjing Hospital of China Medical University, Shenyang, Liaoning, P. R. China; 3 Department of Anatomy, Liaoning Medical University, Jinzhou, Liaoning, P. R. China; UT-Southwestern Med Ctr, United States of America

## Abstract

**Background:**

Abdominal aortic aneurysm (AAA) is a life-threatening disease and its prevalence rate increases with social aging. The degradation of elastic is an important factor in the formation of AAA.

**Methods:**

Adipose derived stem cells (ADSCs) and bone marrow mesenchymal stem cells (BMSCs) were isolated from rats, and identified by Oil red O and alizarin red staining after adipogenesis and osteogenesis induction. In addition, ADSCs were also identified by flow cytometry with CD markers. AAA model in rats was established, and smooth muscle cells (SMCs) were isolated from AAA aortic wall and identified by immunohistochemistry. ADSCs or BMSCs were co-cultured with AAA aortic wall for in vitro experiment, and ADSCs were injected into AAA model for in vivo test. Then orcein staining was used for observing the morphology of elastic fiber, Western blot and real-time PCR were used respectively to detect the protein and gene expression of elastin, gelatinases spectrum analysis was used to detect the activity of matrix metalloproteinase-2 (MMP-2) and MMP-9.

**Results:**

Lots of red lipid droplets were visible by Oil red O staining after adipogenesis induction, and black calcium nodules appeared by alizarin red staining after osteogenesis induction. The results of flow cytometry showed that ADSCs expressed CD44 and CD105, but exhibited negligible expression of CD31 and CD45. SMCs exhibited spindle-like morphology and α-actin protein was positive in cytoplasm. After co-cultured with ADSCs or BMSCs, the elastic fiber recovered normal winding shape, both the gene and protein expression of elastin increased, and the activity of MMP-2 decreased. The in vivo result was similar to that of in vitro.

**Conclusions:**

ADSCs promote the expression of elastin in SMCs and contribute to the reconstruction of elastic fiber, which may provide new ideas for treating AAA.

## Introduction

Aortic aneurysm is a life-threatening disease and its prevalence rate increases with social aging [1]. The localized abdominal aorta expansion, which exceeds more than 50% of the normal vessel diameter, is called abdominal aortic aneurysm (AAA). The tunica media accounts for more than 90% of the abdominal aortic wall, and mainly composed of smooth muscle cells (SMCs) as well as the elastic fiber. Many factors are possibly involved in the formation of AAA, such as chronic inflammation, increase of proteolytic pathways, oxidative stress and the decrease of matrix in the aortic wall [2]. It has also been demonstrated that inflammation may play an important role in aneurysm progression, through the elastin breakdown and SMCs apoptosis mediated by inflammatory cells infiltration and activation of matrix metalloproteinases (MMPs) [3]. It has also been found that AAA could be eased by limiting proinflammatory signals in rats, and the expansion of AAA was positively correlated to inflammatory infiltration [4].

The traditional treatment methods for AAA are medical follow-up and surgical procedure. For large, high fracture risk of AAA, open surgery or endovascular repair is now widely accepted [5]. However, most of the AAA is small and does not reach the standard for operation. On the other hand, surgical mortality and high risk of complications for surgeons and patients are both serious challenge, so we must pay attention to the non-surgical treatment method [6]. It has been reported that some drug receptors could control the development of the AAA in experimental model, and part have been clinically tested. However, the results of clinical trials were not satisfying [7]. With the progress of stem cell research, using stem cells to treat AAA become an increasingly important idea. This kind of cell population is easily separated, and can differentiate into bone, cartilage, heart, etc. under certain conditions, like bone marrow mesenchymal stem cells (BMSCs). Furthermore, ADSCs has some advantages over BMSCs, such as widespread, easily accessible, small trauma, so it becomes a new hotspot of current research. In addition, ADSCs have the effect of the anti-infection and promoting wound healing [8]–[10]. It hasn't been reported so far that ADSCs were used for treating AAA.

In the present study, we have investigated the effect of rat ADSCs on elastin secretion of SMCs in AAA model in vitro or in vivo. Then the study provided a base for the therapy of AAA by ADSCs.

## Materials and Methods

### Ethics Statement

This study was carried out in strict accordance with the recommendations in the Guide for the Care and Use of Animal Research Committee of China Medical University. The protocol was approved by the Animal Research Committee of National Defense Medical Center (IACUC-11-051). All surgery was performed under sodium pentobarbital anesthesia, and all efforts were made to minimize suffering. Animals were housed individually, and were controlled daily by the research team and the animal house staff.

### Isolation, culture and identification of ADSCs

Male SD rats weighing 120 g–150 g were selected and anesthetized by intraperitoneal injection of 3% sodium pentobarbital (30 mg/kg), then disinfected in 75% alcohol. The rat skin was cut along the belly line, muscle tissue was separated and groin subcutaneous fat was cut off. Sterile phosphate buffer solution (PBS) was used to remove blood cells, then adipose tissue was transferred into a small dish and cut into pieces with an eye scissors (volume of debris <1 mm^3^). The debris was digested with 0.1% collagenase solution of type I (Gibco-BRL, Paisley, UK) for 40–50 min at 37°C, then digestion was terminated with DMEM/F12 (DMEM; Gibco-BRL) containing 10% fetal bovine serum (FBS, Gibco-BRL) and centrifuged at 4°C, 1500 rpm for 5 min. The supernatant was discarded, sediment was suspended with DMEM/F12 containing 10% FBS and filtrated with strainer of 200 mesh. Then the filter liquor was centrifuged at 4°C, 1500 rpm for 5 min, and the sediment was resuspended in DMEM-FBS, then transferred into dish and cultured at 37°C with 5% CO_2_ and saturated humidity conditions.

After the third passage, the ADSCs were digested with 0.25% trypsin, and the cell concentration was adjusted to 0.5×10^5^ cells/ml. The cells were then inoculated onto 6-well plates and replaced with inducing medium when cell fusion reached 90%. The adipogenesis inducing medium including 0.5 mM isobutyl-methylxanthine (IBMX), 1 µM dexamethasone, 10 µM insulin, 200 µM indomethacin, 1% antibiotic/antimycotic. The osteogenesis inducing medium including 0.01 µM 1,25-dihydroxyvitamin D3, 50 µM ascorbate-2-phosphate, 10 mM β-glycerophosphate, 1% antibiotic/antimycotic [11]. After 2 weeks of adipogenesis induction, oil red O staining was conducted; after 4 weeks of osteogenesis induction, alizarin red staining was conducted.

In addition, the ADSCs expanded to passage three were examined for surface marker expression using flow cytometry. Briefly, the cultured ADSCs were harvested and washed twice with cold PBS. After centrifugation and removal of the supernatants, the cells were resuspended and incubated with blocking buffer for 30 min at 4°C. Following washing with PBS, the cells were incubated for 30 min at 4°C in the dark with PE -conjugated monoclonal antibodies against CD31、CD44、CD45、CD105 (eBioscience). Flow cytometry analysis was performed with a FACSCalibur cytometer (BD Biosciences) and data were analyzed using CellQuest software.

### Isolation, culture and identification of BMSCs

Male SD rats weighing 120 g–150 g were selected and anesthetized by intraperitoneal injection of 3% sodium pentobarbital (30 mg/kg), then disinfected in 75% alcohol. Then the femur was separated from muscle and fascia, and the two ends were cut off. All the contents of bone marrow were transferred to culture dish by injection syringe containing DMEM/F12 plus 10% FBS, then cultured at 37°C with 5% CO_2_ and saturated humidity conditions. After 3–5 days, the BMSCs grew adherent to the wall while hematopoietic cell suspended in medium because of its characteristic, so changing the medium could remove these nonadherent cells and retain BMSCs. The medium was changed twice weekly and subculture was done when cells reached 80% confluence.

After the third passage, the BMSCs were digested with 0.25% trypsin, and the cell concentration was adjusted to 0.5×10^6^ cells/ml. The cells were then inoculated onto 6-well plates and replaced with inducing medium when cell fusion reached 90%. The adipogenesis inducing medium including basic high-glucose DMEM supplemented with 1.0 µM dexamethasone, 0.5 mM IBMX, 10 µM insulin, 200 µM indomethacin, 10% FBS, 1% penicillin G-streptomycin, and 5.6 mg/L of amphotericin B. The osteogenesis inducing basic high-glucose DMEM supplemented with 100 nM dexamethasone, 10 mM β-glycerophosphate, 0.05 mM ascorbic acid-2-phosphate, 10% FBS, 1% penicillin G-streptomycin, and 5.6 mg/L of amphotericin B [11]. After 2 weeks of adipogenesis induction, oil red O staining was conducted; after 4 weeks of osteogenesis induction, alizarin red staining was conducted.

### AAA model

The experimental AAA model was established by CaCl_2_ infiltration, whereas saline was used in the control group [12]. Rats were first anesthetized by intraperitoneal injection of 3% sodium pentobarbital (30 mg/kg), then sheared and disinfected in the supine position. An incision was made on the abdominal median, and the abdominal cavity was exposed. Then the abdominal aortic below the renal artery and above the iliac artery was dissociated out, and covered by a polyethylene sponge containing 0.75 M CaCl_2_ solution or saline. Meanwhile, the surrounding tissue was protected from the calcium salt infiltration by the sponge encapsulated with sterile rubber strips. After 30 minutes, the sponge and rubber strips were removed and the peritoneal cavity was washed with saline for three times. The incision was sutured layer by layer. Four weeks after the operation, animals were anesthetized and abdominal aorta was isolated for the following assays.

### The co-culture of ADSCs or BMSCs and aortic wall of AAA model

The SD rats of AAA model were anesthetized and abdominal cavity was opened. Then cut off the aorta begin from beneath the diaphragm to the common iliac artery bifurcation, washed the blood with precooling PBS, and co-cultured with ADSCs or BMSCs for 7, 14 and 21 d. The AAA aortic wall cultured alone was used as the control group. Each group had 5 samples, and all experiments were repeated for three times. After that, the AAA aortic wall was fixed in 4% formaldehyde or preserved at −80°C for the follow-up test.

### The primary culture and identification of vascular SMCs

The aorta of AAA model was cut off according to the previous method. Then cut into pieces and transferred them into culture dish. The tissue pieces distributed and adhered to the bottom of dish after culturing for 4 h. Then high glucose DMEM (H-DMEM) plus 20% FBS was added, and cultured at 37°C with 5% CO_2_ and saturated humidity conditions. After 4 days, cells freed out around the tissue and subculture was done when cells reached 80% confluence. After the third passage, the SMCs were conducted with immunohistochemical staining for α-actin.

### In vivo experiments

After the third passage, the ADSCs were adjusted to 4×10^6^ cells/ml and implanted into the artery through the common carotid artery with a 1-ml syringe (0.5 ml per rat). The group that underwent implantation with the ADSCs, saline and PBS were defined respectively as the ADSCs group, sham-operated group and control group. Each group had six rats. After transplantation for 28 d, the abdominal aortic wall was taken out by surgery for gelatin enzyme spectrum analysis, orcein staining and Western blot detection.

### Orcein staining

Orcein staining was used to observe the morphology of elastin. The AAA aortic wall was fixed in 4% formaldehyde for 24 h, dehydrated successively in 10%, 20%, 30% sucrose, then frozed rapidly in liquid nitrogen, embedded with embedding medium and prepared for slices of 8 µm. After placed at room temperature for 1 h, the slices were stained with orcein dye solution for 2 h, then immersed in 95% alcohol for 2 to 3 min and washed off excess dye solution. After dehydration, transparent and seal, the slices were observed with microscope.

### Western blot

After co-cultured with ADSCs or BMSCs for 0, 7, 14 and 21 d in vitro, or injection of ADSCs into AAA model for 28 d, the AAA aortic wall was washed off with PBS, cut into pieces, lysed with RIPA buffer and determined protein concentration by the Bradford method. Equal amounts of protein (40 µg) were used for Western blot analysis with rabbit polyclonal antibodies to anti-elastin (abcam, ab23747, 1∶1000 dilution). Specific antibody binding was detected by horseradish peroxidase-conjugated goat anti-rabbit antibodies and visualized with ECL reagent (Santa cruz) according to the manufacturer's protocol. Antibody to β-actin was used to evaluate protein loading in each lane.

### Gelatin zymogram

Expression of MMP-2 and MMP-9 were assessed using a gelatin zymogram [13]. After co-cultured with ADSCs in vitro or injected with ADSCs in vivo, the AAA aortic wall was isolated and washed with PBS, then cut into pieces in the ice and homogenized (12000 g for 30 min) at 4°C. Protein concentration was determined using a BCA assay, and 50 µg of protein was loaded onto 7.5% Tris-glycine gels with 1 g/L gelatin. Following electrophoresis, gels were washed with distilled water and placed in 2.5% Triton X-100 for washing out SDS, and then incubated at 37°C with gelatinases buffer for 16 h. Gels were stained with 0.1% coomassie blue R-250 and then destained and qualified. A mixture of human MMP-2 and MMP-9 (Chemicon, Temecula, CA, USA) was utilized as standard control. The width and brightness of negative dyed stripe reflected the enzyme activity of gelatin.

### Quantitative real-time polymerase chain reaction (qRT-PCR)

The gene expression of MMP-2, MMP-9 and elastin in SMCs were detected by qRT-PCR after co-culture with ADSCs. The co-culture of SMCs and ADSCs was performed in a transwell system. For that 6×10^4^ ADSCs were seeded onto a polyester membrane transwell-clear insert (Corning, 0.4 µm) while SMCs were seeded onto the bottom of a six-well cell culture plate in the same cell density. After co-cultured for 24, 48 and 72 h respectively, total RNA was isolated from SMCs using the Trizol plus Kit (Life Technologies, Carlsbad, CA, USA) and quantitated using NanoDrop 1000 (NanoDrop, Wilmington, DE). Then the RNA was reverse transcribed into cDNA using PrimeScript RT reagent Kit With gDNA Eraser (TaKaRa). QRT-PCR was performed using SYBR Premix Ex TaqTM II (TaKaRa) on ABI 7500 Real-Time PCR System (Applied Biosystems). Sequences of the primers for MMP 2 were 5′- GATACAGGTGTGCCAAGGTG- 3′ (forward) and 5′- AAAGGGCAAACAAAGCAAAC -3′ (reverse), MMP 9 were 5′- CTGCAGTGCCCTTGAACTAA -3′ (forward) and 5′- TATCCGGCAAACTAGCTCCT-3′ (reverse), elastin were 5′- CATCGGTGGCTTAGGAGTCT-3′ (forward) and 5′- GAAGACCGACACCAGGAACT -3′ (reverse). Sequences of the primers for GAPDH were 5′- AGGCCGGTGCTGAGTATGTC -3′ (forward) and 5′- TGCCTGCTTCACCACCTTCT -3′ (reverse). The PCR amplification was done at: 95°C for 30 sec; 95°C for 5 sec, 60°C for 34 sec for 40 cycles. The relative changes in gene expression data were analyzed by the 2-ΔΔCT method. GAPDH was used as an internal control. Triplicates were run for each sample in three independent experiments.

### Statistical analysis

The results were presented as Mean ± Standard deviation (S.D.) of triplicate determinations. The data were processed using the Statistical Package for the Social Sciences, version 16.0 (SPSS Inc., Chicago, IL, USA). One-way ANOVA was performed for comparison between different groups. Comparisons between groups were conducted using the Tukey Bonferroni test. P values of <0.05 were considered as statistically significant.

## Results

### The morphology of ADSCs in primary culture and differentiation

**Figure 1:**
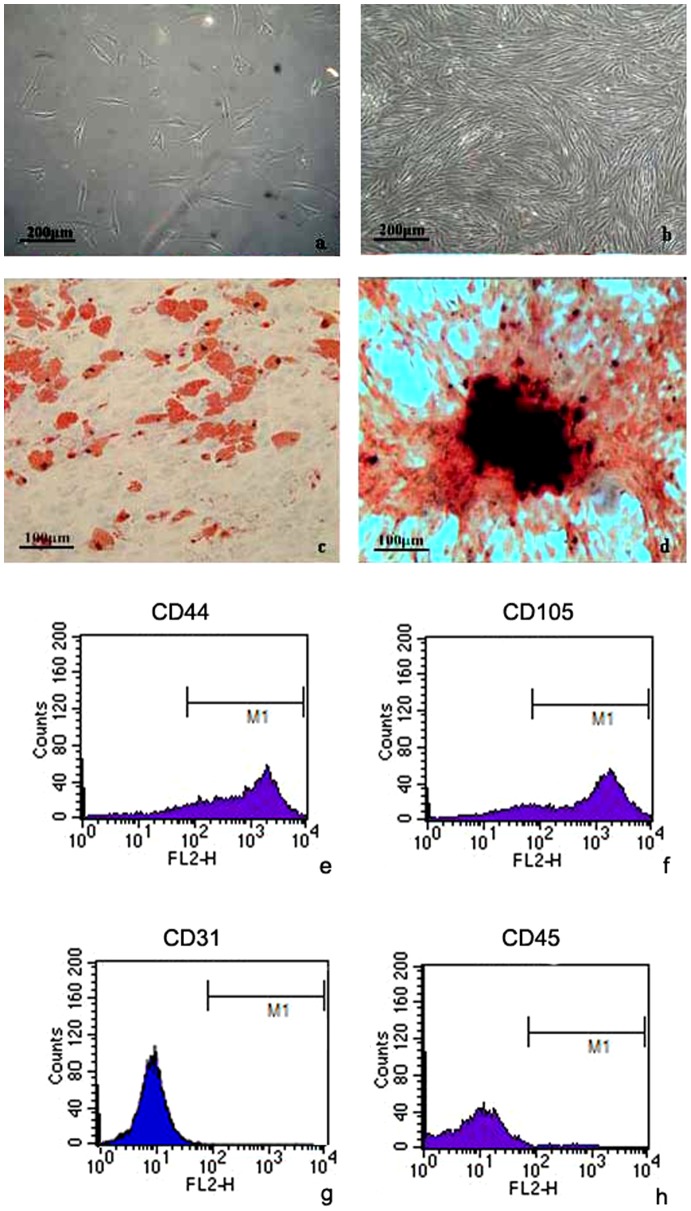
The primary culture and identification of ADSCs. (a) The morphology of ADSCs after plating for 24 h. Bar  = 200 µm. (b) ADSCs achieved a confluence of 80–90% after plating for 7 d. Bar  = 200 µm. (c) ADSCs were stained by Oil red O after adipogenesis induction. Bar  = 100 µm. (d) ADSCs were stained by alizarin red at 14 d of osteogenesis induction. Bar  = 100 µm. (e, f, g, h) Flow cytometry histograms of three passaged ADSCs for CD44, CD105, CD31 and CD45 markers.

During the first day after plating, ADSCs adhered to the plastic surfaces of 6-well plates, as a small population of polygonal or spindle-shaped cells (Fig. 1a). Then ADSCs propagated rapidly in vitro, and showed a homogenous fibroblast-like morphology at 7 d. These cells need to be passaged two to three times in a week, after achieving a confluence of 80–90% (Fig. 1b). After adipogenesis induction, a lot of red lipid droplets were clearly visible by Oil red O staining (Fig. 1c). At 14 d of osteogenesis induction, cells begin to deformation, gathered together, and radiate out. At 28 d, nodular cells mass was visible, as well as red and black calcium, by alizarin red staining (Fig. 1d). Flow cytometry analyses demonstrated that ADSCs expressed CD44 and CD105, but exhibited negligible expression of CD31 and CD45 (Fig. 1e, f, g, h). After statistical analysis, the results were shown as Table 1.

**Table 1:** Cell surface phenotype of ADSCs.

Cell surface markers	Positive rate (%)
CD44	88.05±2.00
CD105	83.82±2.28
CD31	1.45±0.48
CD45	1.03±0.69

Values are the mean ± SD.

### The morphology of BMSCs in primary culture and differentiation

**Figure 2:**
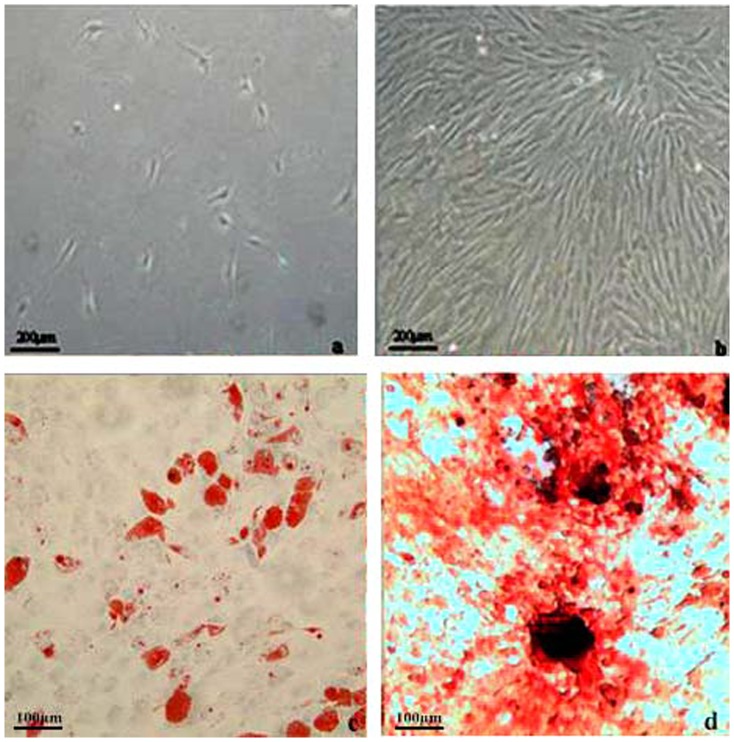
The primary culture and identification of BMSCs. (a) The morphology of BMSCs after plating for 48 h. Bar  = 200 µm. (b) BMSCs achieved a confluence of 80% after plating for 10 d. Bar  = 200 µm. (c) BMSCs were stained by Oil red O after adipogenesis induction. Bar  = 100 µm. (d) BMSCs were stained by alizarin red after osteogenesis induction. Bar  = 100 µm.

Primary cultured BMSCs grew slowly and showed pleomorphism shape (Fig. 2a). At day 10, cells exhibited a fibroblast shape with a unique vortex arrangement at 90% confluence (Fig. 2b). After subculture for three passages or more, BMSC cultures were homogenous populations. At 10 d after adipogenesis induction, cells had morphological changes, gradually in square shape, and red lipid droplets in the cell were visible by Oil red O staining (Fig. 2c). At about 28 d of osteogenesis induction, black calcium nodules appeared by alizarin red staining (Fig. 2d).

### The morphology change of elastic fiber in aortic wall

**Figure 3:**
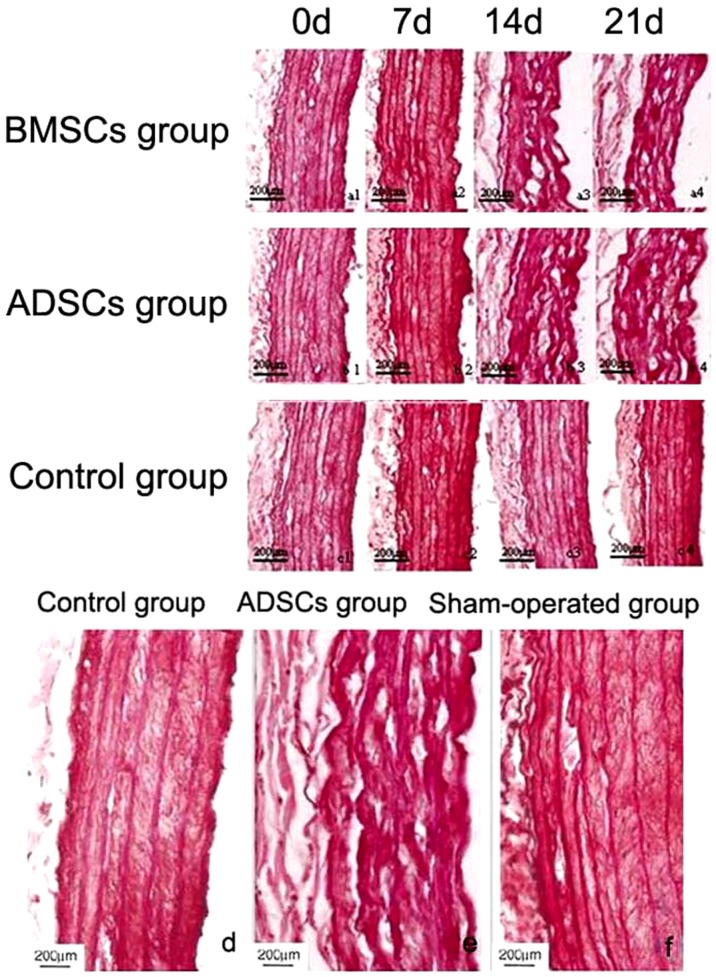
The morphology of elastic fiber after orcein staining. (a, b, c) were the in vitro result and (d, e, f) were the in vivo result. (a1–a4) The morphology of elastic fiber after co-cultured with BMSCs for 0, 7, 14 and 21 d. (b1–b4) The morphology of elastic fiber after co-cultured with ADSCs for 0, 7, 14 and 21 d. (c1–c4) The morphology of elastic fiber in control group. (d, e, f) The morphologies of elastic fiber in control group, ADSCs group and sham-operated group.

After co-cultured with ADSCs or BMSCs for 0, 7, 14 and 21 d in vitro, AAA aortic wall was stained with orcein. The AAA aortic wall without co-culture was used as control group. In co-cultured group, the elastic fiber arranged closely and showed winding shape at 14 d and 21 d, which was similar to the structure of normal aortic wall (Fig. 3a1–a4, 3b1–b4). There was no significant difference between ADSCs and BMSCs group. The results of orcein staining showed that elastic fiber in control group became flat and straight (Fig. 3c1–c4). In vivo experiment, the elastic fiber also appeared wavy shape after injection of ADSCs, while that in control group or sham-operated group was straight and thinner (Fig. 3d, e, f).

### ADSCs contribute to the protein expression of elastin in vitro or in vivo

**Figure 4:**
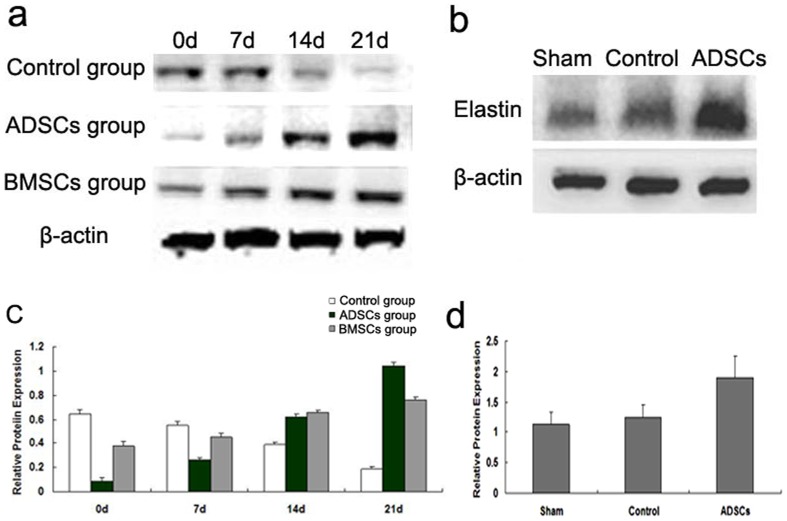
The results of Western blot. (a, c) were the in vitro result and (b, d) were the in vivo result. (a) The protein expression of elastin in control group, ADSCs and BMSCs co-cultured group at 0, 7, 14 and 21 d. β-actin was loading control. (b) The protein expression of elastin in sham-operated group, control group and ADSCs group at 28 d. (c, d) The bar graphs showed mean ± SD of the ratio elastin/β-actin band intensity. P<0.05.

The result of Western blot showed that the protein expression of elastin gradually increased with time after co-cultured with ADSCs or BMSCs compared with that of control group, and that in ADSCs co-culture group was significantly greater than that in BMSCs co-culture group at 21 d (Fig. 4a, c, P = 0.001). The relative protein expression of elastin in ADSCs group was 1.04±0.03 at 21 d, while that in BMSCs group was 0.76±0.02 (Fig. 4c). However, the protein expression of elastin gradually decreased with time in control group (Fig. 4a, c). In vivo experiment, the protein expression of elastin increased significantly in ADSCs group, compared with sham-operated group or control group (Fig. 4b, d, P = 0.011, P = 0.013).

### ADSCs inhibited the expression of MMP-2 and MMP-9 in vitro or in vivo

**Figure 5:**
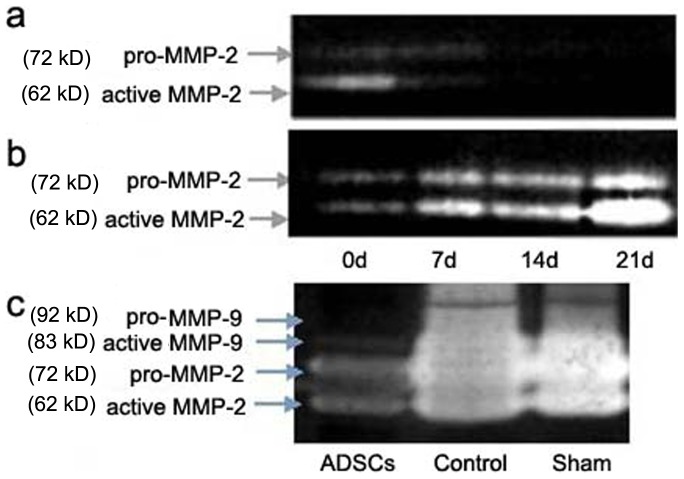
The results of gelatinases spectrum analysis. (a, b) were the in vitro result and (c) was the in vivo result. (a) The expression of pro-MMP-2 and active MMP-2 in ADSCs co-cultured group. (b) The expression of pro-MMP-2 and active MMP-2 in control group. (c) The expression of pro-MMP-9, active MMP-9, pro-MMP-2 and active MMP-2 in ADSCs group, control group and sham-operated group.

The result of gelatinases spectrum analysis showed that the expression of pro-MMP-2 (72KD) and active MMP-2 (62KD) gradually decreased along with time in ADSCs co-culture group (Fig.5a). By contrast, the expression of MMP-2 gradually increased with time in control group, especially the active MMP-2 (Fig. 5b). In vivo experiment, the expression of pro-MMP-9 (92 kD), active MMP-9 (83KD), pro-MMP-2 and active MMP-2 in ADSCs group was less than that in sham-operated group or control group (Fig. 5c), especially MMP-2. Therefore we speculated that the increase of gelatinase activity in this experimental aneurysm was mainly MMP-2, rather than MMP-9, and MMP-2 might play a more important role than MMP-9 in aneurysm formation.

### The morphology of SMCs in primary culture and identification

**Figure 6:**
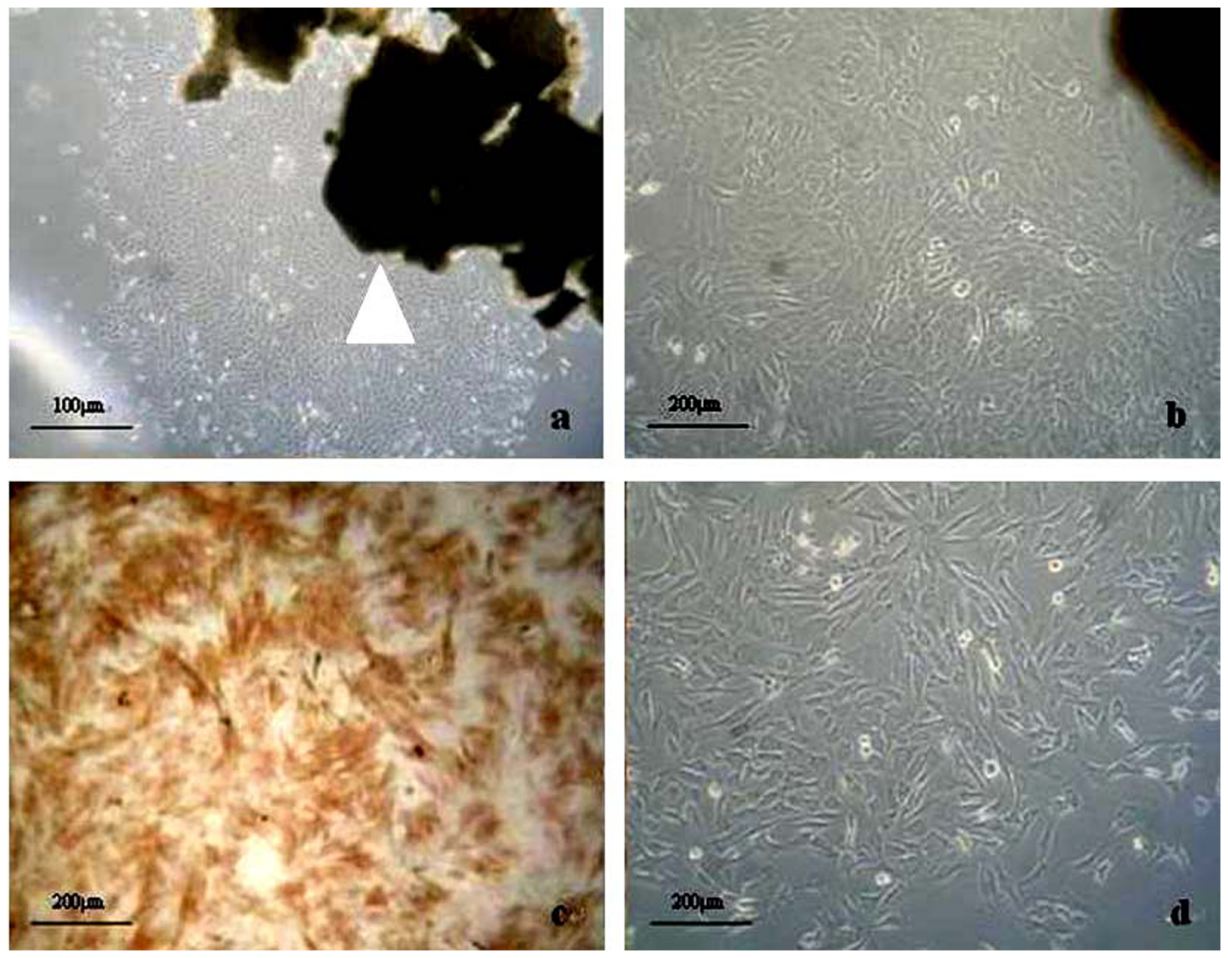
The primary culture and identification of SMCs. (a) At 4 d of primary culture. The arrow points at tissue blocks of AAA aortic wall. Bar  = 100 µm. (b) The morphology of SMCs at 7 d of plating. Bar  = 200 µm. (c) The result of immunohistochemistry for α-actin. Bar  = 200 µm. (d) The morphology of SMCs in normal aortic wall. Bar  = 200 µm.

After 4 d of primary culture, cells were visible around tissue blocks (Fig. 6a). At day 7 of plating, SMCs exhibited the typical spindle-like morphology under optical microscope (Fig. 6b), which was similar to the morphology of SMCs in normal aortic wall (Fig. 6d). The result of immunohistochemistry for α-actin showed brown protein in cytoplasm (Fig. 6c).

### The gene expression of elastin, MMP-2 and MMP-9 in SMCs

**Figure 7:**
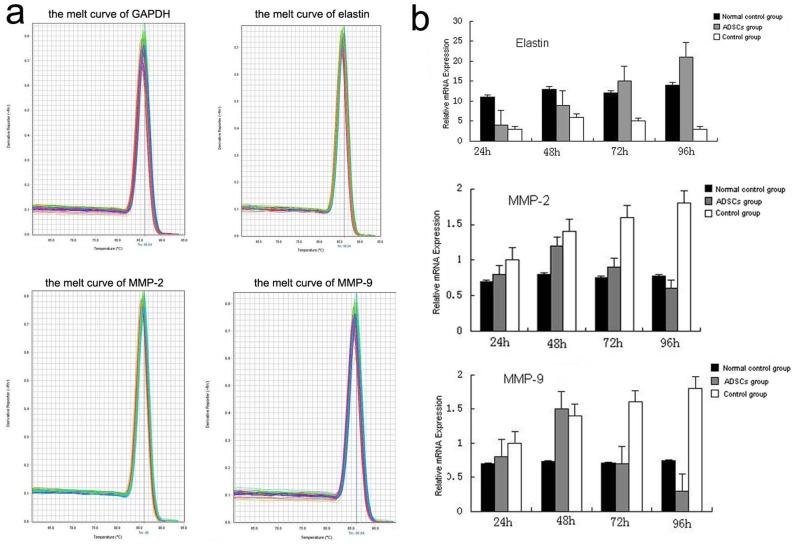
The results of Real-Time PCR. (a) The melting curves of GAPDH, elastin, MMP-2 and MMP-9. (b) The gene expression of elastin, MMP-2 and MMP-9 normal control group, ADSCs group and control group at 24, 48, 72 and 96 h.

QRT-PCR was used to detect the gene expression of elastin, MMP-2 and MMP-9 in SMCs after co-cultured with ADSCs for 24, 48, 72 and 96 h. The result showed that the melting curves of GAPDH, elastin, MMP-2 and MMP-9 were standard single peak, indicating specific amplification products (Fig.7a). In co-culture with ADSCs group, the gene expression of MMP-2 and MMP-9 increased and reached a climax at 48 h, and decreased after that, especially at 96 h. The gene expression of MMP-2 and MMP-9 had no significant difference in normal control group, while that increased with time in AAA control group (Fig.7b, P = 0.009, P = 0.027). However, the gene expression of elastin increased with time in ADSCs co-cultured group (Fig.7b, P = 0.00), while that in normal control group and AAA control group had no significant difference (Fig. 7b, P = 0.052, P = 0.073).

## Discussion

AAA is usually a localized expansion associated with atherosclerosis, and its incidences increase with age [14]. Conventional treatment method is replacing diseased blood vessels with artificial blood vessels. But the accompanying risk factors such as leakage as well as re-intervention rate are higher than that in open surgery [15]. In AAA, the elastic plate of middle lamella of artery was damaged, the number of SMCs decreased, and the invasion of inflammatory cells led to the expansion of arterial wall. The foundation of extracellular matrix remodeling include the steady state of extracellular matrix synthesis in arterial wall of SMCs, the quantity and normal function of SMCs and the protease produced by inflammatory cells, which maintain normal morphology of arterial wall. The causes of aneurysms involved a variety of factors, but if the balance among them was broken, AAA occurred.

According to reports, the decrease of elastin in AAA was related to the excessive secretion of inflammatory cytokines and MMPs, both in the clinical field [16] or in animal models [17]. MMPs are large family, named dependent on zinc and other metal ions, and controlled by tissue inhibitors of metalloproteinase (TIMPs) [18]. The imbalance of excessive elastin degradation is the main cause of aneurysms. Therefore, inhibiting MMPs or increasing the secretion of TIMPs or a combination of both would be a way to limit the development of aneurysms. In fact, MMPs inhibitors applied in vivo have been reported [19]. In recent years, studies have shown that ADSCs could inhibit tumor necrosis factor-α (TNF-α) and promoted the secretion of anti-inflammatory cytokines-interleukin-10 [20]. Whether ADSCs can inhibit secretion of MMPs or not hasn't been reported. In this experiment, we co-cultured AAA aortic wall and ADSCs in vitro, injected ADSCs into rat model of AAA in vivo, then extracted protein from aortic wall and conducted gelatin zymography detection, and the results showed that the activity of MMP-2 and MMP-9 significantly reduced. In addition, SMCs were isolated from AAA aortic wall and co-cultured with ADSCs, the result showed that the gene expression of MMP-2 and MMP-9 also decreased significantly, which demonstrated that ADSCs inhibited the secretion of MMPs and might affect the development of AAA.

Elastin is a kind of extracellular matrix protein and plays an indispensable role in maintaining vascular elasticity. The content of elastin is the most abundant among all proteins in aorta wall [21], and gradually degenerated with age [22]. In animal models, the degradation of elastic plate led to expansion, sclerosis and dysfunction of arterial wall, and ultimately resulted in aneurysm formation or rupture of blood vessels [23]. Although pathological features of aortic aneurysms varied with different positions (ie, thoracic or abdominal aorta), the degradation of elastin was their common characteristics. In this background, it may be a strategy for treating AAA by promoting SMCs secreting elastin and inhibiting proteases meanwhile, including MMPs. In recent years, it has been reported that BMSCs could inhibit inflammatory cytokines and promote the survival of elastin in AAA, but so far there is no report about whether ADSCs can promote the synthesis and secretion of elastin in SMCs. In this experiment, we co-cultured the AAA aortic wall with ADSCs or BMSCs in vitro, then conducted frozen sections and stained with orcein, and found that elastic plate recovered to winding shape again, which was similar to that of normal arterial wall. This phenomenon demonstrated that ADSCs and BMSCs were able to promote the reconstruction of elastic plates. Then we detected the protein expression by Western blot, found that elastin content in ADSCs or BMSCs co-culture group was significant higher than that in control group, and the former was higher that the latter. The findings suggested that both ADSCs and BMSCs could promote the reconstruction of the elastic plate. The in vivo result also verified this conclusion. Therefore it indicated that ADSCs might promote the secretion and synthesis elastin in SMCs, which was likely to provide new ideas for treating AAA. But the related mechanisms in this process are not very clear, which depends on subsequent trials.
